# Causes and Consequences of Innate Immune Dysfunction in Cirrhosis

**DOI:** 10.3389/fimmu.2019.00293

**Published:** 2019-02-25

**Authors:** Katharine Margaret Irvine, Isanka Ratnasekera, Elizabeth E. Powell, David Arthur Hume

**Affiliations:** ^1^Mater Research Institute, Translational Research Institute, The University of Queensland, Brisbane, QLD, Australia; ^2^Department of Gastroenterology and Hepatology, Princess Alexandra Hospital, Brisbane, QLD, Australia; ^3^Faculty of Medicine, The University of Queensland, Brisbane, QLD, Australia

**Keywords:** innate immunity, decompensated chirrosis, infection, monocyte, ascites

## Abstract

Liver cirrhosis is an increasing health burden and public health concern. Regardless of etiology, patients with cirrhosis are at risk of a range of life-threatening complications, including the development of infections, which are associated with high morbidity and mortality and frequent hospital admissions. The term Cirrhosis-Associated Immune Dysfunction (CAID) refers to a dynamic spectrum of immunological perturbations that develop in patients with cirrhosis, which are intimately linked to the underlying liver disease, and negatively correlated with prognosis. At the two extremes of the CAID spectrum are systemic inflammation, which can exacerbate clinical manifestations of cirrhosis such as hemodynamic derangement and kidney injury; and immunodeficiency, which contributes to the high rate of infection in patients with decompensated cirrhosis. Innate immune cells, in particular monocytes/macrophages and neutrophils, are pivotal effector and target cells in CAID. This review focuses on the pathophysiological mechanisms leading to impaired innate immune function in cirrhosis. Knowledge of the phenotypic manifestation and pathophysiological mechanisms of cirrhosis associated immunosuppression may lead to immune targeted therapies to reduce susceptibility to infection in patients with cirrhosis, and better biomarkers for risk stratification, and assessment of efficacy of novel immunotherapies.

## Introduction

Chronic liver injury, most commonly caused by viral infection, alcohol or liver fat accumulation associated with features of the metabolic syndrome, causes activation of resident, and infiltrating immune cells, leading to progressive inflammation and liver fibrosis. Advanced liver fibrosis (cirrhosis) disrupts the normal architecture and function of the liver. Cirrhosis typically has an asymptomatic phase (compensated cirrhosis) followed by a rapidly progressive phase (decompensated cirrhosis) signaled by development of complications of portal hypertension and liver dysfunction (ascites, variceal bleeding, hepatic encephalopathy), which can progress to multi-organ failure known as acute on chronic liver failure (ACLF). Cirrhosis is associated with both systemic inflammation (elevated steady state immune cell activation and circulating inflammatory mediators), which can exacerbate clinical manifestations of cirrhosis such as hemodynamic derangement and kidney injury, and, as disease progresses, immunosuppression, and impaired antimicrobial function, which is associated with increased susceptibility to infection. The immunodeficient state is most marked in the setting of ACLF, which resembles the immunopathology of sepsis, with an initial systemic inflammatory response (cytokine storm) leading to a compensatory anti-inflammatory response that impairs resistance to infection (1). The dynamic spectrum of immunological perturbations that develop in patients with cirrhosis is referred to as cirrhosis associated immune dysfunction (CAID) (2). This review focuses on the pathogenesis and features of innate immune dysfunction that develop in advanced cirrhosis, its role in susceptibility to infection, and recently trialed therapeutic approaches with the potential to alleviate the significant mortality and morbidity associated with infections in these patients.

## Burden and Pathogenesis of Infection in Patients With Decompensated Cirrhosis

Patients with cirrhosis are susceptible to a range of complications, with infections being one of the most clinically important problems, associated with a marked reduction in life expectancy. The rate of bacterial infections is as high as 34% per year in patients with advanced cirrhosis—-in 30–50% of cirrhotic patients, infection is the cause of hospital admission, and a further 15–35% develop nosocomial infections, as compared with 5–7% of the general population (3, 4). Bacterial infections can lead to further decompensation, including variceal hemorrhage and hepatorenal syndrome, and are the main precipitant of ACLF. They account for a 4-fold increase in mortality in hospitalized patients, and a post-infection mortality rate of 28% at 1 month and 63% at 1 year (5, 6). The most common infections in cirrhotic patients are spontaneous bacterial peritonitis (SBP), which alone accounts for more than 30% of infections, urinary tract infections, pneumonia, and soft tissue infections (3, 4, 6).

The majority of infections in patients with cirrhosis are caused by Gram-negative bacteria of intestinal origin, although Gram-positive infections have been associated with severe sepsis in cirrhotic patients in intensive care units (7). Pathological translocation of intestinal bacteria to extraintestinal sites is thus implicated as a major pathogenic mechanism in the development of these infections, especially SBP and bacteremia's. In the early stages of cirrhosis, bacterial translocation contributes to systemic inflammation, with chronic stimulation of immune cells, and elevated levels of circulating inflammatory mediators; which can exacerbate hemodynamic derangements in these patients, lead to further decompensation, and cause tissue damage (2). This chronic, sub-clinical immune activation ultimately leads to immunosuppression, which increases susceptibility to infections, including those due to Gram-positive or bacteria of non-intestinal origin. Overall, the most common Gram negative bacterial infections identified in patients with cirrhosis include *Enterobacteriaceae* such as *Escherichia coli* and *Klebsiella Pneumoniae*, whilst *Staphylococcus aureus*, and *Enterococci* are among the most common Gram positive infections (8, 9). The epidemiology of infections in cirrhosis continues to change with increasing prevalence of multidrug resistant organisms (29–38% increase from 2011 to 2017) (6, 8).

## Pathogenesis of Cirrhosis Associated Innate Immune Dysfunction

Innate immune cells, principally macrophages, monocytes and neutrophils, detect tissue damage, and invading microorganisms, and orchestrate tissue healing and eradication of infection. During chronic liver disease progression they are critical mediators of liver and systemic inflammation, initially responding to so-called damage-associated molecular patterns (DAMPs) released from injured liver cells to initiate and drive progression of fibrosis through activation of hepatic stellate cells and perpetuation of inflammation (10). Systemic perturbations in circulating monocytes and inflammatory cytokines have been reported in pre-cirrhotic and early cirrhotic disease, potentially triggered by ongoing liver inflammation as monocytes traffic through the liver, and, due to the size of the liver, the release of DAMPs and other soluble mediators into the circulation providing constant inflammatory stimuli. Advanced cirrhosis, particularly decompensated disease, is associated with increasing perturbations in gut homeostasis, including microbiome alterations, reduced motility, small intestinal bacterial overgrowth, and increased gut permeability (11); which together lead to increased systemic exposure to gut microbes and microbial products providing further chronic stimulation of innate immune cells. This increase in microbial products may be an important step in the switch from the predominantly proinflammatory immune dysfunction observed in early cirrhosis to a predominant hyporesponsive, immunodeficient phenotype observed in decompensated disease. The inflammatory and immunodeficient phenotypes of cirrhosis associated immune dysfunction are thus the extremes of a dynamic spectrum, both caused by chronic liver inflammation and its systemic consequences.

Mechanistically, the development of this immunodeficient state is driven by innate immune memory–an adaptive property of innate immune cells, particularly monocytes/macrophages whereby prior exposure to microbes or microbial products dictates subsequent responses to stimulation. Whereas, monocyte priming with fungal products typically leads to enhanced inflammatory cytokine production upon re-stimulation (trained immunity), priming with bacterial products, especially lipopolysaccharide (LPS), through toll like receptor (TLR) 4 dampens subsequent responses to stimulation (tolerance) (12, 13). This functional reprogramming is mediated by alterations in intracellular signaling, cellular metabolism and epigenetic changes. LPS tolerance is suggested to be the fundamental mechanism responsible for the immunoparalysis that occurs after Gram negative sepsis, many features of which are also observed in patients with advanced cirrhosis. *In vivo*, innate immune cells are chronically exposed to a dynamic spectrum of stimuli, including microbial products, as the clinical manifestations of disease, especially portal hypertension, worsen. Innate immune function in these patients thus reflects the integration of many stimuli over time. Genetic factors also contribute to increased infection risk in patients with cirrhosis. Common polymorphisms in genes encoding key innate immune pattern recognition receptors including TLR2, TLR4, TLR9, CD14, and NOD2 have been associated with the development of infections in some (14–16) but not all (17) studies. Two polymorphisms in the IL-1 pathway (IL1B and IL1RA) were recently reported to be associated with reduced circulating levels of inflammatory mediators in patients with ACLF (18). As many as 80% of inducible gene expressed by activate human monocytes show heritable variation in their level of expression (19). Such variation has been strongly linked to genetic susceptibility to inflammatory bowel disease (20), which could in turn impact on disordered gut permeability.

## Immunodeficiency in Advanced Cirrhosis

### Liver Immunodeficiency

The liver is an essential organ contributing to immune system homeostasis and defense against infection and is central to both ends of the CAID spectrum. Underlying liver disease drives systemic inflammation, whilst progressive loss of surveillance and synthetic function contributes to hepatic immunodeficiency, and consequently reduced systemic resistance to infection. The liver is the first organ in contact with bacteria and bacterial products originating from the gut via the portal vein, functioning as a filter for gut-derived bacteria that escape surveillance by gut immune cells. Liver resident macrophages, the largest population of tissue macrophages with direct access to the blood stream, are critical for surveillance of blood borne infections, avoiding the systemic spread of microbes and microbial antigens (10). Impaired liver macrophage phagocytic capacity *in vivo* has been demonstrated in patients with cirrhosis (largely of alcoholic etiology) compared to healthy controls, which correlated with liver disease severity, subsequent development of infection and mortality (21, 22). By contrast, the phagocytic capacity of liver macrophages isolated from cirrhotic patients and cultured *ex vivo* did not differ significantly from controls (23). Aside from intact microrganisms, liver resident macrophages contribute to clearance of senescent blood cells (erythrocytes, platelets and neutrophils) and numerous bioactive molecules (including haemopoietic growth factors, hormones (e.g., insulin, glucocorticoids and parathyroid hormone) cytokines and chemokines, food and luminal antigens, toxins (both microbial and xenobiotic), clotting factors/fibrin, lipoproteins, prostanoids, and immune complexes) (24). Liver spillover and increased levels of these endocytic substrates into the circulation contributes to a systemic pathogenic milieu.

Increased circulating levels of agents that are normally cleared by the liver is accompanied by the loss of products normally produced by the liver, including factors that comprise the humoral component of innate immunity. Both hepatocytes and liver macrophages synthesize soluble factors, such as complement and soluble pattern recognition receptors (e.g., lipopolysaccharide binding protein, soluble CD14) that are essential for effective systemic immune responses and resistance to infection. In response to inflammation, the liver produces acute phase proteins, such as Mannose Binding Lectin (MBL), C-reactive protein (CRP), hepcidin, fibrinogen, and proteinase inhibitors, which participate in innate immune responses and control tissue damage and repair. Reduced production of complement and other proteins such as ficolins and MBL that contribute to bacterial recognition and killing, is commonly observed in cirrhotic patients and linked to the development of infections and other clinical outcomes (25–30). Reduced production of CRP, which is routinely used as a biomarker of infection in the general population, in response to bacterial infection has also been reported in patients with cirrhosis (3, 31). Serum bactericidal activity is impaired in cirrhotic patients, and correlated with low complement levels (25). One of the most common blood abnormalities associated with liver disease is thrombocytopenia, which may be a consequence of both dysregulated platelet clearance and decreased production of thrombopoietin. Platelets and the clotting cascade are an important component of bacterial recognition. The mechanisms and the impacts of platelet deficiency, on pathogen clearance have been reviewed recently (10, 32).

While the extent of and mechanisms underlying liver macrophage dysfunction in cirrhotic patients have not been widely investigated, it was recently demonstrated in mice that gut bacterial translocation induced type 1 interferon expression in the liver, conditioning myeloid cells to produce high levels of IL-10 upon Listeria infection, impairing antibacterial immunity and leading to a loss of infection control (33). A prominent liver interferon signature was also observed in patients with cirrhosis, and liver myeloid cells showed increased IL-10 production after bacterial infection (33). In cirrhosis, systemic spread of gut-derived bacteria and bacterial products is also facilitated by portosystemic shunts, which form as a result of portal hypertension and divert portal blood from the liver to the systemic circulation.

### Circulating Innate Immune Cells

Circulating innate immune cells play a key role in resistance to blood borne infections, which are common patients with cirrhosis (34), and are also recruited to tissues to effectively combat infections. Although the impact of immune phenotype and function observed *ex vivo* on bacterial clearance function *in vivo* is difficult to investigate in patients with chronic liver disease, Ashare et al. took advantage of the transient bacteremia that occurs following tooth-brushing, and demonstrated prolonged bacteremia in patients with cirrhosis compared to healthy controls and patients with pulmonary disease, which correlated with disease severity (Child Turcotte Pugh (CTP) and Model for End Stage Liver Disease (MELD) scores) (35). This acutely impaired bacterial clearance suggests functional impairments in circulating neutrophils and/or monocytes, the principal innate immune effector cells, *in vivo*.

### Neutrophil Phenotype and Function in Cirrhosis

Neutrophils are a key component of the innate immune system that play vital roles in defense against infection but have also been shown to contribute to the development of systemic inflammation and organ failure in sepsis. Circulating neutrophils exhibit limited microbicidal activity in the steady state; they require priming by endogenous inflammatory mediators or microbial agents to exert maximal functionality, with upregulation of endothelial adhesion molecules required for recruitment to damaged or infected tissues. Key neutrophil antimicrobial activities include extrusion of neutrophil extracellular traps, phagocytosis, production of reactive oxygen species (ROS, termed the respiratory burst), and the release of antimicrobial proteases and other mediators via degranulation.

The most consistent findings in relation to circulating neutrophil function in patients with cirrhosis are diminished phagocytic capacity and/or elevated ROS production at steady state (36–42), which correlate with serum pro-inflammatory cytokines and markers of bacterial translocation, increase with disease severity measures (36, 38, 41), and correlate with the development of infections and mortality (38, 41). In a prospective study of 62 patients, baseline neutrophil phagocytic capacity and resting ROS production predicted the development of infection, organ dysfunction and mortality at 90 days and 1 year (41). Notwithstanding the elevated resting ROS production; impaired neutrophil killing of intracellular bacteria (43) and reduced capacity to mount an augmented oxidative burst response is observed in patients with cirrhosis in general (43, 44) and in patients with active infections (36). In patients with decompensated alcoholic cirrhosis, neutrophil antimicrobial activity and microbicidal protein release was severely impaired. Bactericidal activity could be restored *ex vivo* by the TLR7/8 agonist CL097, which potentiated signaling through the formyl peptide receptor and myeloperoxidase release (45). Garfia et al. reported selective impairment of Phospholipase C-, but not Calcium/Protein Kinase C-dependent activation of the burst response in patients with cirrhosis (44), whilst Rajkovic et found depleted intracellular glutathione in cirrhotic patient neutrophils, suggesting reduced ability to detoxify ROS and resulting oxidant stress may contribute to impaired neutrophil function (43). Impaired neutrophil phagocytosis in cirrhosis has also been associated with reduced responsiveness to Tuftsin, a natural tetrapeptide that can stimulate neutrophil phagocytosis (42).

Several studies have demonstrated that serum/plasma from patients with cirrhosis can transmit the elevated ROS and impaired phagocytosis phenotype to healthy neutrophils (38, 46–48). In one study, depletion of serum LPS restored neutrophil dysfunction (38), suggesting impairments may be reversible. However, serum inhibitory factors have not associated with neutrophil impairment in all cirrhotic cohorts (42, 43).

Impaired neutrophil endothelial adhesion and chemotaxis *in vitro* (46, 49, 50) as well as mobilization to an aseptic inflammatory site *in vivo* (skin blister) (48, 49), have also been demonstrated in patients with cirrhosis. Impaired neutrophil migration and phagocytosis of heat-killed *E. coli in vivo* in patients with cirrhosis was further reduced in patients with previous episodes of bacterial infection compared to non-infected patients (49). In this study, circulating neutrophil complement receptor 3 (CR3), an integrin that mediates neutrophil endothelial adhesion and binding to complement-opsonised bacteria, was elevated in cirrhotic patients compared to healthy controls, but reduced in patients with a previous bacterial infection. Granulocyte colony stimulating factor (G-CSF) treatment increased transendothelial migration of neutrophils from cirrhotic patients *in vitro*, suggesting this factor may be able to increase neutrophil recruitment to infected sites (50). Elevated expression of other neutrophil adhesion molecules, including LSEL [CD62L (50)] and ITGAM (CD11B) (37), which may contribute to alterations in neutrophil recruitment *in vivo*, have also been reported.

Together, the available evidence supports the conclusion that neutrophils in patients with cirrhosis are chronically activated, exhibiting high resting ROS production but are impaired in their ability to traffic to sites of infection and to mount effective antimicrobial responses. These perturbations are linked to persistent *in vivo* stimulation with microbial products or other inflammatory mediators, and the degree of neutrophil dysfunction increases with disease severity, and likely contributes to increased susceptibility to infection. Cirrhosis-associated neutrophil dysfunction may be reversible as several studies have reported improved function *in vitro* with interventions such as LPS-depletion (38), TLR7/8 agonism (45, 50), G-CSF (50), and GM-CSF treatment (46). Notably, most, though not all, investigations of neutrophil function in cirrhosis have been conducted in patients with alcoholic liver disease. Alcohol consumption is known to elevate neutrophil ROS production and impair phagocytosis, even in people without chronic liver disease (51), and alcohol use is a significant contributor to gut permeability and bacterial translocation in cirrhotic patients (52).

### Monocyte Phenotype and Function in Cirrhosis

In addition to augmenting tissue macrophage pools via recruitment to inflammatory sites, circulating monocytes are important innate immune effector cells in their own right, and also initiate and regulate the development of adaptive immunity, via antigen presentation and the production of immunoregulatory cytokines. Although there is clearly evidence of increased monocyte infiltration of the diseased liver (10), there is little evidence of a profound monocytosis in liver disease; most focus has been on changes in function and differentiation. Human blood monocytes have been broadly classified into 3 subsets based on CD14 and CD16 expression; “classical” CD14high/CD16- monocytes (comprising ~80% of peripheral blood monocytes) that express high levels of chemokine (C-C motif) receptor (CCR)2, non-classical CD14+CD16+ monocytes, which preferentially express the chemokine (C-X3-C motif) receptor (CX3CR)1 and intermediate CD14HighCD16+ monocytes. Functionally, classical monocytes exhibit strong phagocytic capacity, whilst non-classical monocyte subsets, in particular the intermediate subset, have been designated pro-inflammatory (53, 54). Detailed transcriptome and enhancer profiling of these “subsets” indicated that they represent a differentiation series, in which the intermediate population expresses intermediate levels of every transcript that distinguishes classical and non-classical monocytes (55). Alterations in monocyte subsets, in particular an increase in intermediate and/or non-classical monocytes, are frequently observed in infectious and inflammatory diseases, and have been associated with clinical outcomes (53). Modest elevations in non-classical monocytes, that have limited phagocytic capacity and high production of inflammatory cytokines, have been reported even in early stages of chronic liver disease in some (56, 57), but not all (58) studies, with the most prominent perturbations suggested to occur in patients with cirrhosis. CD16+ inflammatory monocytes have also been suggested to preferentially accumulate in the liver (56, 57).

Monocyte surface marker expression, especially microbial pattern recognition receptors (since increased activation by LPS and other microbial stimuli is postulated to be responsible for high pro-inflammatory cytokine levels in cirrhosis) has been investigated in a number of studies, with inconsistent results. Xing et al. reported higher monocyte expression of TLR4 (that recognizes LPS from Gram-negative bacteria, among other ligands) and TNF production in patients with hepatitis B viral infection (HBV) and ACLF compared to healthy controls (59). By contrast, selective down-regulation of TLR4-dependent immune responses, which was restored by Gram-negative-targeted antibiotic therapy, was proposed to contribute to impaired monocyte function and infection risk in cirrhosis, suggesting a role for Gram negative gut microbes in suppressing peripheral TLR4 responses (60). Other studies suggest a stimulatory role for Gram-positive bacterial components in monocyte dysfunction in cirrhosis. Riordan et al. reported that monocyte TLR2, but not TLR4, expression was increased in patients with cirrhosis, and significantly correlated with serum TNF, whilst *in vitro* TNF production in response to a Gram-positive bacterial infection was blunted (61). Manigold et al. also reported elevated TLR2, but reduced TLR4 expression, in cirrhotic patients, which strongly correlated with serum levels of LPS (62). In addition to TLR expression, reduced monocyte expression of CCR2 (a key chemokine receptor for monocyte recruitment to infected or inflamed tissue sites) has been reported, regardless of disease stage (57, 58), and we found elevated expression of the adhesion receptor LSEL (CD62L) in patients with decompensated cirrhosis (58).

The most consistent finding with regard to monocyte phenotype in cirrhosis is a reduction in HLA-DR expression (58, 63–66), although increased expression has also been reported (57, 67). Reduced HLA-DR expression, which likely compromises antigen presentation and the development of adaptive immune responses, is one of the hallmarks of LPS tolerance, the immunosuppressed state in which monocytes become refractory to further stimulation with LPS and other microbial stimuli. Monocyte HLA-DR expression appears to diminish with advancing disease. A step-wise reduction in HLA-DR expression was observed in HBV-infected patients with non-cirrhotic chronic liver disease, compensated cirrhosis and ACLF (59). HLA-DR expression was modestly reduced in our cohort of patients with NAFLD, and significantly reduced in patients with HCV, with the most pronounced reduction in patients with decompensated cirrhosis (58). In ACLF, expansion of HLA-DRLow monocytes was associated with poor clinical outcomes, with significantly lower HLA-DR expression in patients who died compared to those who survived (68). Similarly, the percentage of HLA-DR+ monocytes was significantly reduced patients with decompensated cirrhosis, both with and without ACLF (with alcohol as the commonest etiology in both cohorts), compared to healthy controls; and low HLA-DR expression was associated with death from ACLF (65). In a longitudinal study of critically ill cirrhotic patients, low HLA-DR expression at baseline was associated with mortality, and reduced further over time in non-survivors, compared to stable or increasing expression in survivors (69), suggesting impaired HLA-DR expression is reversible. Longitudinal alterations in HLA-DR were a stronger predictor of survival than measures of liver insufficiency (MELD score) or organ failure (Sequential Organ Failure Assessment (SOFA) score). There is limited evidence of a functional consequence of reduced HLA-DR, but one study reported attenuated antigen-specific T cell responses in cirrhotic patients, associated with a high frequency of CD14+/HLADR- monocytes (64). Nevertheless, *ex vivo* monocyte-derived dendritic cells from cirrhotic patients had a similar capacity to upregulate co-stimulatory molecules and stimulate expansion of antigen-specific T cells compared to healthy controls, further suggesting recovery of HLA-DR expression is possible (70).

Monocyte production of pro-inflammatory cytokines in response to microbial activation is key to innate immune defense against infection, as these cytokines enhance antimicrobial functions. Reduced monocyte production of pro-inflammatory cytokines (in particular TNF and IL-6) in response to LPS stimulation is another hallmark of the LPS tolerant immunosuppressed state in sepsis and other severe inflammatory conditions. In early chronic liver disease as well as compensated and decompensated cirrhosis, elevated spontaneous and/or LPS-induced monocyte TNF production have been identified (57, 67, 68). Impaired inflammatory cytokine (TNF, IL-6, IL-1, IL-12) production only becomes apparent in late stage ACLF (68). Similarly, plasma from patients with acutely decompensated, but not compensated, cirrhosis suppressed LPS-induced TNF production by healthy monocytes (71). This suppressive effect was mediated by increased plasma Prostaglandin E2 (PGE2) in acutely decompensated cirrhosis related to low levels of albumin, which regulates PGE2 bioavailability (71). In our cohort of patients with chronic liver disease, LPS-induced TNF production was not impaired in patients with decompensated cirrhosis overall, but was significantly reduced in patients who died during 6 months follow up and correlated with time to death (58).

Monocyte bactericidal capacity depends on effective phagocytosis and the action of phagolysosomal enzymes as well as the production of ROS. Although monocyte bacterial killing has not been directly investigated in chronic liver disease or cirrhosis, there is some evidence that monocyte antimicrobial functions are impaired in these patients. Elevated resting ROS production, and reduced ability to respond to microbial challenge with augmented ROS production (oxidative burst), correlated with advancing disease, has been reported (30). In patients with HCV or NAFLD, we found monocyte capacity to mount an oxidative burst response was significantly reduced in patients with cirrhosis as well as in patients with chronic liver disease but without advanced fibrosis (58). By contrast Bruns et al. reported impaired neutrophil but not monocyte oxidative burst capacity in cirrhotic patients (predominantly alcoholic liver disease) (36). Monocyte phagocytic capacity was severely impaired in patients with ACLF, related to the expansion of a poorly phagocytic HLA-DRLow immature myeloid population (68). We found monocyte phagocytic capacity for *E. coli* bioparticles was negatively correlated with ALT, suggesting a relationship with liver injury and inflammation, but was not significantly impaired in patients with compensated or decompensated cirrhosis (58). Like cytokine production, impaired phagocytic capacity may be a late event in the course of decompensated disease.

Together, the available evidence suggests that chronically activated monocytes contribute to the systemic inflammatory state in patients with chronic liver disease by production of inflammatory cytokines, but significant impairment in monocyte function, especially reduced HLA-DR expression and stimulated cytokine production, progressively occurs in decompensated cirrhosis, and is associated with clinical events and outcomes. Although the pathophysiological mechanisms underlying features of monocyte suppression have not been elucidated, high levels of LPS and anti-inflammatory cytokines (such as IL-10) and reduced levels of interferon γ have been suggested to contribute (63). Given the diversity of bioactive molecules that spillover into the circulation from the diseased liver, it seems unlikely that any single stimulus is entirely responsible. Both HLA-DR expression and cytokine expression may be reversible, however; interferon γ therapy restored monocyte function and resulted in clearance of sepsis in a small cohort of critically ill patients (72).

### Peritoneal Innate Immune Phenotype and Function in Cirrhosis

Ascites is the most common complication of decompensated cirrhosis, and SBP is the most common infection in these patients, with a prevalence of up to 50% in patients admitted to hospital, and mortality of ~30% (3, 4). SBP is considered to be mainly caused by gut-derived microbes, as a result of increased intestinal permeability and bacterial translocation. The main identifiable causes of SBP are Gram negative, enteric microbes (especially *E. coli*) and Gram-positive Streptococci. However, SBP is diagnosed by an elevated ascites fluid neutrophil count (>250/ml) because the rate of culture negativity in the diagnostic microbiological analysis of ascites samples is high (up to 60% (11)), even where there are clinical signs of infection, necessitating empirical antibiotic treatment. The efficacy of currently recommended empirical antibiotic therapy was reported to be 83% in community-acquired infections, but low in health-care acquired infections (40%) (73), highlighting the need to identify adjunct approaches to prevent infections in this high-risk population. Unlike other tissues, including the liver, ascites fluid is relatively accessible, facilitating investigations into innate immune function at this key site of infection. It should be noted, however, that ascites fluid and the cells it contains only comprise part of the picture, as the omentum also plays essential roles in peritoneal defense (74).

The presence of microbial products, including LPS, bacterial peptides and, particularly, bacterial DNA in ascites fluid, even in the absence of overt infection, is a consistent finding (75–80). The presence of bacterial DNA was associated with similar cytokine profiles in ascites fluid to that seen in the presence of active infection (81). These findings suggest peritoneal immune cells, like circulating cells, in patients with decompensated cirrhosis are chronically exposed to microbes and/or microbial products. Separate studies revealed an association between low peritoneal macrophage HLA-DR expression and the presence of bacteria in both uninfected and infected patients (76, 82), the latter study also demonstrating resolution with antibiotic treatment. Ascites macrophage HLA-DR expression did not correlate with circulating monocyte HLA-DR (76), suggesting, not surprisingly, that the peritoneal microenvironment shapes macrophage phenotype and function. The underlying etiology of cirrhosis significantly affected ascitic immune cell subsets and cytokine levels, with lower leukocyte numbers and higher inflammatory cytokine levels in patients with alcoholic cirrhosis compared to HCV (83). Other studies have identified further differences between ascites macrophages and donor-matched circulating monocytes, including relatively increased HLA-DR and cytokine (TNF, IL-6, IL-10) expression and stronger responses to e*x vivo* LPS and Candida stimulation in ascites macrophages, whereas monocytes produced significantly more IL-12 and IL-1β (84, 85).

Both factors present in the peritoneal microenvironment and cell intrinsic differences may contribute to the differences observed between peritoneal and circulating monocytes/macrophages. Murine peritoneal macrophages can be subdivided into mature resident cells, which are capable of self-replication, and monocyte-derived macrophages which slowly replace the resident population and acquire a differentiated phenotype (86). The mature resident peritoneal macrophages have a unique gene expression profile, including high expression of genes encoding phagocytic receptors (e.g., Vsig4, Timd4, and Marco) (87). Similarly, two phenotypically and functionally distinct macrophage populations can be identified in human patient ascites fluid; a highly phagocytic population that expresses high levels of CD14, HLA-DR, and Complement Receptor for Immunoglobulin (CRIg, encoded by the VSIG4 gene) and resemble murine resident peritoneal macrophages, and a poorly phagocytic population, which express CCR2 and resemble murine peritoneal monocyte-derived macrophages (88). The relative proportions of CRIgHi and CCR2+ macrophages varied between patients, and within patients over time. A high proportion of CRIgHi macrophages and macrophage phagocytic capacity significantly correlated with lower disease severity (MELD score) (88). A similar relationship between monocyte-derived populations and lower disease severity was also inferred based on CD14 (85), which is more highly-expressed on the CRIgHi population. The reduced ascites macrophage CD14 and HLA-DR expression and low macrophage phagocytic capacity reported in patients with SBP compared to sterile ascites (82) probably reflects dilution by the CD14Low monocyte-derived macrophage population in the setting of infection. Compared to peritoneal macrophages from non-cirrhotic controls, ascites macrophages from cirrhotic patients without overt infection had a higher oxidative burst response to microbial stimulation, and lower phagocytic capacity (89).

In addition to being a biomarker of bacterial translocation, bacterial DNA in ascites fluid is also an innate immune stimulus. Because a variety of pathogens use the cytosol for replication, it is under surveillance by DNA-sensing pattern recognition receptors, including the Absent in Melanoma 2 (AIM2) inflammasome, that induces a broad inflammatory response, and the cGAS-STING axis, that drives antiviral immunity by inducing type 1 interferons. Lozano-Ruiz et al. reported elevated expression of AIM2, which specifically induces inflammasome formation in response to double stranded DNA, and constitutive caspase 1 activation and IL-1β production in blood and ascites fluid macrophages from patients with decompensated cirrhosis, in the absence of infection, which correlated with disease severity (CTP score) (90). Bacterial DNA-stimulated inflammasome activation *ex vivo* did not require a priming signal, suggesting ascites macrophages were pre-activated i*n vivo*. Although, suggesting a role for AIM2, these data are also consistent with DNA-dependent activation of the NLRP3 inflammasome, downstream of the cGAS-STING pathway that has recently been reported (91).

Neutrophils are also a significant cellular component of ascites fluid in patients with cirrhosis, especially in the presence of infection, and they mirror some of the systemic deficiencies discussed above. Ascites neutrophil oxidative burst capacity in response to Phorbol Myristate Acetate (PMA) stimulation was significantly impaired in patients with SBP compared to sterile ascites, and this resolved with antibiotic treatment in patients with culture-negative, but not culture-positive, SBP (82). The respiratory burst in neutrophils from patients with cirrhotic ascites was impaired compared to malignant ascites and inhibitory factors were identified in ascites fluid independent of complement factor 3 [C3, which is known to be deficient in cirrhotic ascites fluid (92, 93) and predispose to SBP (94)]. Engelmann et al. (95) compared blood and peritoneal neutrophil function in patients with decompensated cirrhosis and non-cirrhotic controls and found impaired phagocytosis and oxidative burst activity upon *E. coli* challenge in cirrhotic ascites compared to peripheral blood, whereas non-cirrhotic ascites had higher phagocytic activity but equally suppressed oxidative burst (52). Ascites neutrophil function could be partially restored after incubation with autologous plasma, underscoring the role of inhibitory factors in ascites fluid in impaired neutrophil function.

In addition to cellular antimicrobial function, ascites fluid has been reported to have poor opsonising activity (29, 96), with low levels of C3 reported in particular (92, 93), that have also been associated with the development of infection at this site.

## Biomarkers of Immune Dysfunction With in Cirrhosis

Since the transition from innate immune activation to dysfunction seems to be a key pathophysiological mechanism contributing to the development of infection, immune function-based biomarkers may have utility for risk stratification, identification of patients who may benefit from immunotherapy, and assessing outcomes of therapeutic interventions in patients with cirrhosis. Although perturbations in pro- or anti-inflammatory biomarkers have been associated with clinical outcomes in decompensated cirrhosis (97–99), these do not necessarily reflect changes in functional immunity and direct assessment of cellular function may be required. Functional innate immune responses, including increased resting neutrophil ROS production and phagocytic capacity, have been associated with the development of infections and survival (38, 41), suggesting potential utility as indicators of susceptibility, but they may be difficult to translate into clinical practice. Monocyte HLA-DR expression appears to diminish progressively with advancing disease, and longitudinal changes in monocyte HLA-DR expression were strongly associated with mortality (mostly from sepsis) in critically ill patients with cirrhosis, as has been demonstrated in non-cirrhotic sepsis (69). The utililty of monocyte HLA-DR expression as a biomarker of infection risk at earlier disease stages remains to be clarified. The development of standardized methods of flow-cytometric assessment of HLA-DR expression may facilitate translation to the clinic in the future (100). In an integrated approach to assessing innate and adaptive immune function, reduced interferon production in response to combined innate and adaptive immune stimulation with the TLR7 agonist R848 and anti-CD3 (Quantiferon platform) was associated with the development of infections in patients with decompensated cirrhosis (101). With further validation Quantiferon may be a promising biomarker in cirrhosis, as this platform has been successfully adapted for clinical use (for the detection of latent tuberculosis infection).

## Immunotherapeutic Approaches to Reduce Susceptibility to Infection in Cirrhosis

Since gut bacterial translocation is considered to be the main source of infection in patients with decompensated cirrhosis, infection prevention is mainly based on selective intestinal decontamination using orally administered poorly absorbed antibiotics such as norfloxacin (targeting Gram-negative *Enterobacteriacea* and *Enterococci*). Antibiotic prophylaxis carries the risk of development of antibiotic resistance, limiting therapy and increasing morbidity and mortality (102). Prophylaxis is thus limited to patients at very high risk of developing infections, defined by clinical criteria (3). A variety of novel adjunct approaches to antibiotic prophylaxis have been explored, including strategies targeting intestinal bacterial overgrowth and/or abnormal gut microbiota (non-selective intestinal decontamination (Rifaximin), probiotics, fecal microbiota transplantation), intestinal motility (prokinetics, beta-blockers) and barrier function (bile acids, beta-blockers) (102). Strategies to improve intestinal homeostasis would be anticipated to beneficially impact systemic immune function, via reducing chronic stimulation that leads to immunosuppression as well as reducing exposure to potentially infectious organisms. Many antibiotics, including those used prophylactically in patients with decompensated cirrhosis, also exert direct effects on the immune system, although the underlying mechanisms are not well-defined (103).

In addition to gut-targeted approaches, there is also emerging interest in immunotherapeutic approaches to reduce susceptibility to infection in decompensated cirrhosis. However, given the co-existing spectrum of inflammation and immunodeficiency in cirrhosis, timing of immunotherapy appears critical. In the setting of ACLF, therapies to limit hepatic and systemic inflammation that contribute to multiorgan failure may be relevant in the early stages of decompensation, whereas immune boosting approaches may be beneficial in later stages where there is prolonged immunosuppression and high risk of secondary infections. Immunotherapeutic approaches for the treatment and prevention of infection in ACLF have recently been comprehensively reviewed (104). The potential of prophylactic immunotherapy to prevent infections, which are a major precipitant of ACLF, in relatively stable decompensated cirrhosis warrants further investigation. The literature reviewed herein suggests significant immunosuppression is only present in a subset of patients with “stable” decompensated cirrhosis; those at risk of poor outcomes, including infections. Identification and application of appropriate therapies requires better understanding and improved assessment of immune function to identify patients most likely to benefit from immunotherapy. Two strategies that have been the subject of recent clinical trials, albumin and granulocyte colony stimulating factor treatment, are discussed below.

### Albumin Supplementation

Albumin is a 67 kDa protein synthesized exclusively in the liver and released to the intravascular compartment. It has multiple functions such as binding and transport of substances, regulation of endothelial function, antioxidant and scavenging properties and regulation of inflammatory responses (105). In chronic liver disease, serum albumin level is reduced due to defective synthesis and altered structurally and functionally due to post-transcriptional modification, hindering its ability to perform its physiological functions (106, 107). Albumin modifications that affect its binding capacity have been reported in cirrhotic patients, and correlated with liver disease severity measures, the development of complications and survival (108). Oxidized serum albumin, that is commonly elevated in patients with cirrhosis, was also recently shown to directly trigger an inflammatory response in peripheral blood mononuclear cells (97). Intravenous albumin is commonly used to regulate blood volume in patients with decompensated cirrhosis, and has been proven to prevent renal failure (109). Patients with low serum or ascitic fluid albumin have also been shown to be at increased risk of infection (110) but the immunoregulatory effects of albumin administration are still unclear. A large (*n* = 1,818) randomized controlled trial in patients with severe sepsis found no improvement in outcomes in patients administered albumin in addition to a crystalloid volume expander compare to crystalloid solution alone, however this study did not achieve the target serum albumin level of >30 g/L (111).

Patients with decompensated cirrhosis were recently demonstrated to have high levels of plasma PGE2, which impaired LPS-stimulated TNF production in healthy monocytes *ex vivo*. Monocyte function could be restored by albumin supplementation via reducing PGE2 bioavailability (71). On the basis of this finding, albumin supplementation has been investigated as an immunomodulatory therapy in decompensated cirrhosis. In a recent single arm feasibility study (*n* = 79) daily administration of 20% human albumin solution to hospitalized patients with ACLF and low serum albumin levels for 14 days (or until discharge or death) increased serum albumin to the target level of >30 g/L [since albumin < 30 g/L was the only clinical characteristic that predicted suppressed monocyte responses in *in vitro* studies (71)] without significant safety concerns (112). Patient plasma significantly suppressed macrophage LPS-stimulated TNF production compared to healthy plasma, and restoration of serum albumin to >30 g/L failed to alter plasma PGE2 levels, but did increase albumin PGE2-binding capacity and restore TNF production to similar levels observed in healthy plasma (113). A randomized controlled trial of albumin administration to reduce infections in ACLF has commenced recruitment. In another study, albumin co-administration for 3 days following SBP diagnosis (50 ml 20% Human Albumin Solution) only modestly increased serum albumin, but significantly reduced serum and ascitic fluid cytokines (IL-6 and TNF) and LPS, compared to patients receiving antibiotic therapy alone (*n* = 15 per group) (114). Albumin treatment also reduced ascites fluid TNF in patients without SBP (114). In addition to short-term administration to acutely decompensated patients, the efficacy of long-term albumin administration in patients with ascites has recently been investigated in a randomized, multi-site trial (115). Four hundred forty patients with decompensated cirrhosis (uncomplicated ascites) received standard medical care or standard care plus albumin (40 g twice weekly for 2 weeks then weekly for up to 18 months). Overall 18-month survival was significantly higher in patients who received albumin compared to standard care (115). Given its excellent safety profile and these promising results, albumin appears to have potential as a prophylactic treatment in patients with decompensated cirrhosis, possibly targeted to patients with demonstrable immunosuppression, however the optimum dose and treatment duration remains to be clarified, with consideration of the cost-effectiveness of therapy and the availability of human albumin in hospitals.

### Granulocyte Colony Stimulating Factor (G-CSF) and Stem Cell Therapy

G-CSF, a potent cytokine that mobilizes CD34+ haematopoietic stem cells from the bone marrow, and autologous stem cell therapy have been investigated for their potential to improve outcomes in patients with decompensated cirrhosis, including ACLF. Mobilization or delivery of hematopoietic stems cells, which have the capacity to differentiate into multiple cell lineages, is postulated to promote liver regeneration. A renewed supply of leukocytes may also overcome the immune paralysis that contributes to the development of infections. G-CSF may also improve the function of existing immune cells, as G-CSF improved neutrophil transendothelial migration *in vitro* (50). Several randomized controlled trials (RCTs) of G-CSF therapy have been conducted in patients with cirrhosis. Most employ a dose regime established to mobilize HSC from the bone marrow (~5 daily doses of 5 ug/kg), though some studies continued to dose weekly for an extended period. One RCT in patients with ACLF demonstrated significant improvement in liver disease severity measures, reduced development of multi-organ failure and other complications (including sepsis) and a >2-fold 60-day survival rate after 30 days G-CSF therapy (*n* = 23–24 per group). G-CSF therapy significantly increased peripheral leukocyte counts and hepatic CD34+ cells (116). G-CSF treatment was further shown to increase circulating and intrahepatic myeloid dendritic cells, CD4 and CD8 T cells; and to reduce CD8 T cell IFNγ production (117). Although IFNγ is a key factor known to restore monocyte immunosuppression *in vitro* and in sepsis (72), innate immune phenotype and function were not investigated. In this cohort, G-CSF was administered prior to the onset of sepsis and the authors suggested that early identification of patients and commencement of G-CSF treatment could prevent the development of sepsis and multiorgan failure (116). G-CSF therapy was also demonstrated to improve liver function and 3-month survival, with significantly fewer patients dying of sepsis in the treatment compared to the control group, in patients with HBV-associated ACLF (*n* = 27–28 per group) (118). In another study, treatment of patients with decompensated cirrhosis with G-CSF and erythropoietin for 1 month reduced liver disease severity (CTP score) and improved survival at 12 months follow-up compared to placebo, and significantly fewer patients in the treatment arm developed sepsis during follow up (*n* = 26–29 per group) (119).

An RCT conducted in a cohort of decompensated cirrhotic patients (excluding ACLF, *n* = 21–23 per group) assessed the impact of 3 monthly G-CSF administration for 1 year, with and without Growth hormone (GH, postulated to promote liver regeneration), compared to standard care. Transplant free survival at 12 months was significantly improved in both groups of G-CSF-treated patients, associated with decreased measures of liver disease severity and a significant reduction in the odds of developing a bacterial infection (including ~88% reduction in the odds of developing sepsis). GH treatment did not show any additional benefit in reducing infections or in survival (120). In contrast to these promising results, G-CSF treatment for 5 consecutive days or G-CSF treatment followed by 3 doses of autologous CD133+ hematopoietic stem cells in patients with compensated cirrhosis did not improve liver disease severity (MELD score, the primary outcome), and there was evidence to suggest G-CSF treatment, with or without stem cells, was associated with an increased frequency of adverse events compared to standard care (*n* = 26–28 per group) (121). In a study by Sharma et al. patients with non-viral decompensated cirrhosis received a hepatic artery infusion of autologous CD34+ hematopoietic stem cells (mobilized by 3 consecutive days G-CSF treatment) or standard care (*n* = 22–23 per group). Stem cell therapy significantly increased serum albumin levels at 1 month but this was not sustained at 3 months, and there was no difference in survival between the treatment arms (122). Overall, G-CSF and stem cell therapy appear to be promising approaches to improve liver function and reduce the burden of infections in patients with ACLF, but may not be applicable in non-ACLF decompensated cirrhosis as efficacy has not been consistently demonstrated and one study raised safety concerns.

## Conclusion

**Figure 1:**
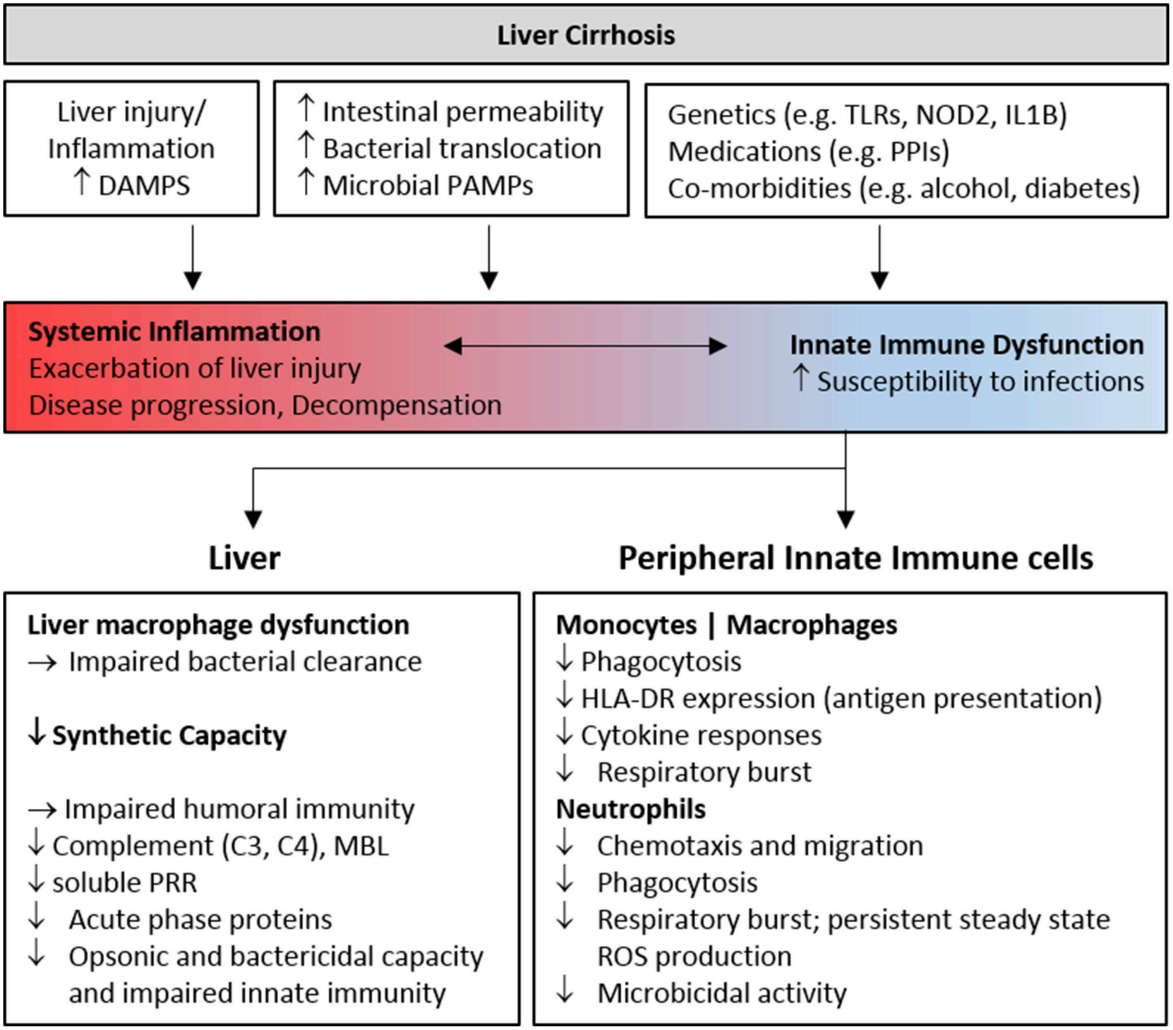
Features of innate immune deficiency in advanced cirrhosis. Host and microbial inflammatory mediators resulting from liver injury and gut bacterial translocation initially activate innate immune cells, contributing to inflammation and the development, and progression of liver cirrhosis. Chronic stimulation progressively perturbs innate immune sensing and surveillance functions, contributing to infection risk in patients with decompensated cirrhosis. Genetic and environmental factors also contribute to immune dysfunction. The features of cellular and humoral innate immune deficiency commonly identified in cohorts of patients with advanced cirrhosis are summarized. DAMP, Damage Associated Molecular Pattern; PAMP, Pathogen Associated Molecular Pattern; PRR, Pattern Recognition Receptor; PPI, Proton Pump Inhibitor; MBL, Mannose Binding Lectin; TLR, Toll-like receptor.

Infections are very common in patients with decompensated cirrhosis, they precipitate further decompensation and multi-organ failure (ACLF) and are therefore associated with high morbidity and mortality. Prevention of bacterial infections in these patients is crucial to improve outcomes, however current approaches are limited to antibiotic therapy, which is associated with antibiotic resistance and increased infections with non-classical pathogens (6, 8, 9). The identification of risk factors contributing to the development of infections in this vulnerable population, including impaired immune resistance to infection, may provide novel adjunct therapeutic approaches to minimize the burden of infections, improve outcomes and minimize antibiotic resistance. With regards to innate immunity, the first line of defense against infection, whilst chronic activation is apparent in pre-cirrhotic and compensated cirrhotic disease, functional impairment becomes progressively apparent in decompensated cirrhosis, potentially associated with increased gut permeability and exposure to gut-derived microbes and microbial products. The most commonly identified features of innate immune dysfunction in decompensated cirrhosis are summarized in Figure 1. Whilst defects in core immunological functions that contribute to infection resistance (phagocytosis, ROS production, cytokine production) have consistently been reported, actual killing capacity for relevant pathogens has rarely been investigated. This is potentially important as it was recently demonstrated in mice that reduced cytokine production as a result of microbial product-induced tolerance did not correlate with bacterial killing *in vivo* (123). Moreover, increased understanding of other antimicrobial functions (e.g., autophagy), may lead to novel therapeutic approaches.

Increased resting neutrophil ROS production and reduced monocyte HLA-DR expression with disease progression have been consistently linked to outcomes and may aid in risk stratification or provide useful biomarkers of therapeutic efficacy, at least in research settings. However, identification of universal features of cirrhosis associated immunodeficiency, and the clinical stage at which they occur, is hampered by the dynamic interplay between factors driving systemic inflammation and immunosuppression in cirrhosis, and the variability in patient cohorts that have been studied. Many studies do not distinguish between compensated and decompensated cirrhosis, whilst others focus exclusively on patients with ACLF. In addition to disease stage, medications (e.g., steroids, proton pump inhibitors, non-selective beta blockers), underlying liver disease etiology and associated lifestyle factors also impact intestinal permeability and immune function (e.g., diabetes, alcohol). Immunotherapeutic approaches to prevent infections in patients with decompensated cirrhosis are only beginning to be explored, and trials to date have only reported short term outcomes. Given its excellent safety profile and the recent preliminary evidence for efficacy, albumin administration is a promising strategy. Improved understanding of the pathophysiology and natural history of the spectrum of immune activation and immunodeficiency in the course of decompensated cirrhosis may enable the identification of targeted preventative immunotherapies in the future.

## Author Contributions

KI wrote the paper. IR contributed to literature review and writing. EP and DH contributed to writing and critically reviewed the paper.

### Conflict of Interest Statement

The authors declare that the research was conducted in the absence of any commercial or financial relationships that could be construed as a potential conflict of interest.
