# Corrigendum: Causes and Consequences of Innate Immune Dysfunction in Cirrhosis

**DOI:** 10.3389/fimmu.2019.00818

**Published:** 2019-04-09

**Authors:** Katharine Margaret Irvine, Isanka Ratnasekera, Elizabeth E. Powell, David Arthur Hume

**Affiliations:** ^1^Mater Research Institute, Translational Research Institute, The University of Queensland, Brisbane, QLD, Australia; ^2^Department of Gastroenterology and Hepatology, Princess Alexandra Hospital, Brisbane, QLD, Australia; ^3^Faculty of Medicine, The University of Queensland, Brisbane, QLD, Australia

**Keywords:** innate immunity, decompensated chirrosis, infection, monocyte, ascites

In the original article, we neglected to include the funder “Equity Trustees.”

The funding statement has therefore been revised as follows:

“KI, IR, and DH are grateful for research funding support from the Mater Research Foundation and Equity Trustees, Australia.”

The authors apologize for this error and state that this does not change the scientific conclusions of the article in any way. The original article has been updated.

